# Supplementary material for: Co-Occurrence of Wing Deformity and Impaired Mobility of Alates with Deformed Wing Virus in Solenopsis invicta Buren (Hymenoptera: Formicidae)
Source: Insects. 2023 Sep 27;14(10):788. doi: 10.3390/insects14100788 (PMC10607916; doi:10.3390/insects14100788)
Supplement: Supplementary file 1 [file insects-14-00788-s001.zip › insects-2593642-supplementary.pdf]

Co-occurrence of Wing Deformity and Impaired Mobility of Alates with Deformed Wing Virus (DWV) in *Solenopsis invicta* Buren (Hymenoptera: Formicidae)

Godfrey P. Miles[a], Xiaofen F. Liu[a], Esmaeil Amiri[b], Michael J. Grodowitz[a], Margaret L. Allen[a], and Jian Chen[a,1]

Author affiliations: [a]Biological Control of Pests Research Unit, USDA-ARS, Stoneville, MS 38776, USA; [b]Mississippi State University, Delta Research and Extension Center, 82 Stoneville Road, Stoneville, MS 38776.

Supplementary Materials

Table S1. Information of *S. invicta* colonies used in this study.

Data Set S1A-H. Results of Sanger sequencing of *S. invicta* samples.

Videos. Description of eight videos depicting both DW and NW male and female alates from *S. invicta* lab and field colonies (https://doi.org/10.5281/zenodo.8357376).

Figure S1. Phylogenetic tree based on sequences from samples of *S. Invicta* and positive control of *A. mellifera* with DWV against deformed wing virus sequence fragments on which the sequencing primer sets were located.

Colony Label	Abbreviated	Collected or In Situ	Collection Date	Location County	GPS
RIFA-WASH-08-(4)	2021	Collected	11/16/2021	Washington	33.16 N, 90.90 W
RIFA-WASH-08-(4)	2021B	Collected	11/16/2021	Washington	33.16 N, 90.92 W
RIFA-WASH-01	1	Collected	6/2/2022	Washington	33.16 N, 90.92 W
RIFA-WASH-02	2	Collected	6/2/2022	Washington	33.16 N, 90.92 W
RIFA-WASH-03	3	Collected	6/2/2022	Washington	33.16 N, 90.92 W
RIFA-WASH-04	4	Collected	6/2/2022	Washington	33.16 N, 90.92 W
RIFA-WASH-05	5	Collected	6/2/2022	Washington	33.16 N, 90.92 W
RIFA-WASH-06	6	Collected	6/2/2022	Washington	33.16 N, 90.92 W
RIFA-WASH-07	7	Collected	6/2/2022	Washington	33.16 N, 90.92 W
RIFA-WASH-08	8	Collected	6/2/2022	Washington	33.16 N, 90.92 W
RIFA-WASH-09	9	Collected	6/2/2022	Washington	33.16 N, 90.92 W
RIFA-WASH-10	10	Collected	6/2/2022	Washington	33.16 N, 90.92 W
RIFA-WASH-11	11	Collected	6/2/2022	Washington	33.16 N, 90.92 W
RIFA-WASH-12	12	Collected	6/2/2022	Washington	33.16 N, 90.92 W
RIFA-WASH-13	13	Collected	6/2/2022	Washington	33.16 N, 90.92 W
RIFA-WASH-14	14	Collected	6/2/2022	Washington	33.16 N, 90.92 W
RIFA-WASH-15	15	Collected	6/2/2022	Washington	33.16 N, 90.92 W
RIFA-WASH-1B	1B	Collected	8/30/2022	Washington	33.16 N, 90.92 W
RIFA-WASH-2B	2B	Collected	8/30/2022	Washington	33.16 N, 90.92 W
RIFA-WASH-3B	3B	Collected	8/30/2022	Washington	33.16 N, 90.92 W
RIFA-WASH-4B	4B	Collected	8/30/2022	Washington	33.16 N, 90.92 W
RIFA-WASH-5B	5B	Collected	8/30/2022	Washington	33.16 N, 90.92 W
RIFA-WASH-1C	1C	Collected	1/27/2023	Washington	33.16 N, 90.92 W
RIFA-WASH-2C	2C	Collected	1/27/2023	Washington	33.16 N, 90.92 W
RIFA-WASH-3C	3C	Collected	1/27/2023	Washington	33.16 N, 90.92 W
RIFA-WASH-4C	4C	Collected	1/27/2023	Washington	33.16 N, 90.92 W
RIFA-WASH-5C	5C	Collected	1/27/2023	Washington	33.16 N, 90.92 W
RIFA-WASH-Greenville-1	G-1	Collected (new queen)	7/22/2021	Washington	33.35 N, 91.04 W
RIFA-WASH-Greenville-2	G-2	Collected (new queen)	7/23/2021	Washington	33.35 N, 91.04 W
RIFA-WASH-NBCL-1	NBCL-1	In Situ	8/31/2022-to-11/10/2022	Washington	33.43 N, 90.92 W
RIFA-WASH-NBCL-2	NBCL-2	In Situ	8/31/2022-to-11/10/2022	Washington	33.43 N, 90.92 W
RIFA-WASH-NBCL-3	NBCL-3	In Situ	8/31/2022-to-11/10/2022	Washington	33.43 N, 90.92 W
RIFA-WASH-NBCL-4	NBCL-4	In Situ	8/31/2022-to-11/10/2022	Washington	33.43 N, 90.92 W
RIFA-WASH-NBCL-5	NBCL-5	In Situ	8/31/2022-to-11/10/2022	Washington	33.43 N, 90.92 W
RIFA-WASH-NBCL-6	NBCL-6	In Situ	8/31/2022-to-11/10/2022	Washington	33.43 N, 90.92 W
RIFA-WASH-NBCL-7	NBCL-7	In Situ	8/31/2022-to-11/10/2022	Washington	33.43 N, 90.92 W
RIFA-WASH-NBCL-8	NBCL-8	In Situ	8/31/2022-to-11/10/2022	Washington	33.43 N, 90.92 W
RIFA-WASH-NBCL-9	NBCL-9	In Situ	8/31/2022-to-11/10/2022	Washington	33.43 N, 90.92 W
RIFA-WASH-NBCL-10	NBCL-10	In Situ	8/31/2022-to-11/10/2022	Washington	33.43 N, 90.92 W
RIFA-WASH-NBCL-11	NBCL-11	In Situ	8/31/2022-to-11/10/2022	Washington	33.43 N, 90.92 W
RIFA-WASH-NBCL-12	NBCL-12	In Situ	8/31/2022-to-11/10/2022	Washington	33.43 N, 90.92 W
RIFA-WASH-NBCL-13	NBCL-13	In Situ	8/31/2022-to-11/10/2022	Washington	33.43 N, 90.92 W
RIFA-WASH-NBCL-14	NBCL-14	In Situ	8/31/2022-to-11/10/2022	Washington	33.43 N, 90.92 W

Colonies G-1 and G-2 were initiated using new queens collected on July 21, 2021, in a parking lot of Nelco Cineplex, Greenville, Mississippi. The apparatus method used to set up the new colonies was described by [34].

Supplementary Materials: Data Set S1A. Results of Sanger sequencing of samples of *S. invicta* ants from Figure 3., showing positive results for replicative form of DWV: adult workers from colony 8, (pooled, *n*=10), worker pupae, colony 10, (pooled, *n*=5), and deformed wing (DW) male alates, colony 8, (pooled, *n* = 5), showing positive detection for the replicative form of DWV using a tag-primer that was incorporated into a fragment of the RNA-dependent RNA polymerase (RD-RP) gene during the reverse transcriptase step. Samples were ten-times diluted. Additional sequence is from a NW male alates showing the replicative form of the DWV, which is at two-times dilution.

Adult workers from colony 8, (pooled, *n*=10) **[% Identity; Top Accession]**
>Workers_DWV-B23_DWV-10Xdilu-22Mar23_A06_2023-03-22_3.ab1; from Contig 3 (237 bases) [98.00%; MH069505.1]
GAGCACATGTTTAGGTGGATGTTTTTGAACCCATGTTTTAAGAAAGTAGCAGTCTGTAACGTCCGCCACTTTACAGTATTTCCTGATTTATCCTGATCCGTAAATTCCATCTTATATTGTGAAAAGAATTTTCCTATCGTCACGGCGT
TAAACTTATCAATCATGTTATCGCTAACATTCATGATAAGATCATCGCCATAACAGACAAGAACAACATTTTGAGAGAACTCGGACAAA
Worker pupae, colony 10, (pooled, *n*=5)
>Pupae_DWV-B23_DWV-10Xdilu-22Mar23_B06_2023-03-22_3.ab1 (439 bases) [99.17%; MH069505.1]
CGGCTACCTCTACGGCTCCACTCGGTACTCTGACTCCCGCACATGCCGCTCTAACGCCCTTCTACCGACTCCTTTTCCAGGTTAGCTAGAAACACAGGTCTAGTTGGATGTTTTAAGAACCCATGTTTTAAGAAAGTAGCAGTCTGT
AACGTCCGCCACTTTACAGTATTTCCTGATTTATCCTGATCCGTAAATTCCATCTTATATTGTGAAAAGAATTTTCCTATCGTCACGGCGTTAAACTTATCAATCATGTTATCGCTAACATTCATGATAAGATCATCGCCATAACAGA
CAAGAACAACATTTTGAGAGAACTCGGACAAAGGCAAATCAGTAATACCTAACCAAGCTAACCTAATTAACAAACAATTTGAAATTGTATTCAATATGTCCGTTATTGGAGAACCTGATGGACCACGGTGCGCAGGCTATAAGG
Deformed wing (DW) male alates, colony 8, (pooled, *n* = 5) (423 bases); 99.46%; MH069505.1]
>DW-alates_DWV-B23_DWV-10Xdilu-22Mar23_C06_2023-03-22_3.ab1; from Contig 3
CTTAAGGCAGGGGAAACGGCGCGCTGACTCTCTAACGCCCCAATTCGTCGTTCCTTCTACCGAGACCTTGTCCAGGTTAGCTAGAAACACAGGTCTAGTTGGATGTTTTAAGAACCCATGTTTTAAGAAAGTAGCAGTCTGTAAC
GTCCGCCACTTTACAGTATTTCCTGATTTATCCTGATCCGTAAATTCCATCTTATATTGTGAAAAGAATTTTCCTATCGTCACGGCGTTAAACTTATCAATCATGTTATCACTAACATTCATGATAAGATCATCGCCATAACAGACAA
GAACAACATTTTGAGAGAACTCGGACAAAGGCAAATCAGTAATACCTAACCAAGCTAACCTAATTAACAAACAATTTGAAATTGTATTCAATATGTCCGTTATTGGAGAACCTGATAGACCACAGTGCGC

Normal wing male (NW), colony 14, (single alate) (373 bases) (male)[98.66%; MG831204.1]
>14-NW-M-1B_Tag_2XD_DWV-rep-17Feb23_F06_2023-02-21_2.ab1 (two-times dilution)
ATGGTTGGGTATTACTGATTTGCCTTTGTCAGAGTTCTCTTAAAATGTTGTTCTTGTCTGTTATGGCGACGATCTTATCATGAATGTTAGTGATAACATGATTGATAAGTTCAACGCCGTGACGATAGGAAAATTCTTTTCACAATA
TAAGATGGAATTTACGGATCAGGATAAATCAGGAAATACTGTAAAGTGGCGGACGTTACAGACTGCTACTTTCTTAAAACATGGGTTCTTAAAACATCCAACTAGACCTGTGTTTCTAGCTAACCTGGACAAGGTCTCGGTAGAA
GGAACGACGAATTGGACTCATGCTCGAGGATTGGGTCGTCGTACAGCAACCATAGAAAATGCTAAACAAGCGTTAGAGTTA

Supplementary Materials: Data Set S1B. Results of Sanger sequencing of multiple samples from workers, a NW female alate and a Queen of *S. invicta* showing positive detection for DWV using subvariant-A-specific primers.

B

Ants (adult workers) from various DWV-positive colonies **[% Identity; Top Accession]**

>1_DWV-A-R_Ants-13Oct2022_A04_2022-10-13_3.ab1 (123 bases) [100%; OP889268.1]

ACACGCAAATTATCAGTCCACACAAGATCAAAGTCAGCAATATGTTCATGACGCTTACTACACCACGTAGCTATATATTTCTTCTGTTTTCCAGTTATATAACCTTCATCCACAAGACAAAGC

>2_DWV-A-F_Ants-13Oct2022_B03_2022-10-13_3.ab1 (131 bases) [98.36%; MF409157.1]

ATTCTACGCTACGTGGTGTAGTAAGCGTCATGAACATATTGCTGACTTTGATCTTGTGTGGACTGATAATTTGCGTGTGTTAAGCGCGTATGCGCATGAACGTTCATCTTCAACTCGGCTTTCTACGGAAA

>2_DWV-A-R_Ants-13Oct2022_B04_2022-10-13_3.ab1 (109 bases) [100%; OP889268.1]

TATCAGTCCACACAAGATCAAAGTCAGCAATATGTTCATGACGCTTACTACACCACGTAGCTATATATTTCTTCTGTTTTCCAGTTATATAACCTTCATCCACAAGACA

>3_DWV-A-F_Ants-13Oct2022_C03_2022-10-13_3.ab1 (128 bases) [98.43%; MF409157.1]

ATATAGCTACGTGGTGTAGTAAGCGTCATGAACATATTGCTGACTTTGATCTTGTGTGGACTGATAATTTGCGTGTGTTAAGCGCGTATGCGCATGAACGTTCATCTTCAACTCGGCTTTCTACGGAA

>3_DWV-A-R_Ants-13Oct2022_C04_2022-10-13_3.ab1 (134 bases) [98.48%; MG831202.1]

GCATACGCGCTTACACACGCAAATTATCAGTCCACACAAGATCAAAGTCAGCAATATGTTCATGACGCTTACTACACCACGTAGCTATATATTTCTTCTGTTTTCCAGTTATATAACCTTCATCCACAAGACAA

>4_DWV-A-F_Ants-13Oct2022_D03_2022-10-13_3.ab1 (120 bases) [99.16%; MF409157.1]

CGTGGTGTAGTAAGCGTCAGTGAACATATTGCTGACTTTGATCTTGTGTGGACTGATAATTTGCGTGTGTTAAGCGCGTATGTGCATGAACGTTCATCTTCAACTCGGCTTTCTACGGAA

>4_DWV-A-R_Ants-13Oct2022_D04_2022-10-13_3.ab1 (111 bases) [99%; KY451706.1]

GTCCACACAAGATCAAAGTCAGCAATTTGTTCATGACGCTTACTACACCACGTAGCTATATATTTCTTCTGTTTTCCAGTTATATAACCTTCATCCACAAGACAACGGAAT

>6_DWV-A-R_Ants-13Oct2022_F04_2022-10-13_3.ab1 (116 bases) [98.84%; OP889268.1]

TATCAGTCACTACTACACCGTATCATCGATCAGCACATATGTTCATGACGCTTACTACACCACGTAGCTATATATTTCTTCTGTTTTCCAGTTATATAACCTTCATCCACAAGACA

(+) control - honeybee

>7_DWV-A-R_DWV-3OCT22_G05_2022-10-04_3.ab1 (120 bases) [100%; OP889268.1]

ACACACGCAAATTATCAGTCCACACAAGATCAAAGTCAGCAATATGTTCATGACGCTTACTACACCACGTAGCTATATATTTCTTCTGTTTTCCAGTTATATAACCTTCATCCACAAGAC

>7_DWV-A-F_DWV-3OCT22_G02_2022-10-04_3.ab1 (135 bases) [98.41%; MF409157.1]

AAACATTATAGCTACGTGGTGTAGTAAGCGTCATGAACATATTGCTGACTTTGATCTTGTGTGGACTGATAATTTGCGTGTGTTAAGCGCGTATGCGCATGAACGTTCATCTTCAACTCGGCTTTCTACGGAATC

DWV-A Contigs

>Contig1 11 reads, 180 bases [98.82%; MF409144.1]

CGYTTTGTCTTGTGGATGAAGGTTATATAACTGGAAAACAGAAGAAATATATAGCTACGTGGTGTAGTAAGCGTCATGAACATATTGCTGACTTTGATCTTGTGTGGACTGATAATTTGCGTGTGTTAAGCGCGTAT
GCGCATGAACGTTCATCTTCAACTCGGCTTTCTACGGAAGGAG

>Contig2 6 reads, 108 bases [97.03%; KX373899.2]

AGTCTATGGTGGTCTTCATCATCGGGATTTCTTGGAGCTTGAGGCTCTGTATATTGACATTCCACTGATGAACGCCGAACTTGTTCATGCGCTTCAATCTCCGARMAA

>Queen_B8_DWV-11-30-2022_A04_2022-11-30_2.ab1; from Contig 2 (286 bases) [100%; OL803824]

GCATACCTAAATTTGATATGATGGAAATCTTTCAACATCTTAGGACTACATTCAGCAATAGGAATATCATTCTCACAATGCTTGCATCCTCGCTTCTTCTCTTCACTCGCTTTACATTCAATCAAAACATTTCTACGCCGATAAATAGC
TTCCATAGCAATACGATCAAACCTCGGGAAAGGCTTATTCGTATTGTATATGAATATTTCCGGGTTATATCGCATTTTCTTACCTTCTAAATCAGCTTTAGGAGGAGAAAGCACGATAGGGGAATGGACCTGGAAAA

>Queen_F6_DWV-11-30-2022_A03_2022-11-30_2.ab1; from Contig 2 (334 bases) [99%; MH267695.1]

ATCCTCTATAGGCCGAAGAAAGTCATATACAATACGAATAATCCTTTCCCGAGGTTTGATCGTATTGCTATGGAAGCTATTTATCGGCGTAGAAATGTTTTGATTGAATGTAAAGCGAGTGAAGAGAAGAAGCGAGGATGCAAG
CATTGTGAGAATGATATTCCTATTGCTGAATGTAGTCCTAAGATGTTGAAAGATTTCCATCATATCAAATTTAGGTATGCACATGACGTATGTAATTCCGAGACTACATGGTCTGAATGGATGACGTATAATGAATTTCTTGAATG
GATAACTCCTGTGTATATGGCTAACCGTCGTAAGGCGAATGAAG

>NW-F_DWV-A-R_DWV-11-30-2022_E02_2022-11-30_2.ab1; from Contig 1 (118 bases) [100%; OP889268.1]
CGAACATTATCAGTCCACACAAGATCAAAGTCAGCAATATGTTCATGACGCTTACTACACCACGTAGCTATATATTTCTTCTGTTTTCCAGTTATATAACCTTCATCCACAAGACAAA
>NW-F_DWV-A-F_DWV-11-30-2022_E02_2022-11-30_2.ab1; from Contig 1 (147 bases) [88%; MF409157.1]
GGCAGCCGTCAGCCCTATATATGTCTACGA

Supplementary Materials: Data Set S1C. Results of Sanger sequencing of multiple samples from pooled workers, DW and NW male alates (single and pooled), and pupae of *S. invicta* showing positive detection for DWV using subvariant-A-specific primers.

C

>1_DWV-A-F_Worker Ants-20Oct2022_A01_2022-10-20_1.ab1 (110 bases) [97.17%; MF409157.1]
GCGTCATGAAACATATTGCTGACTTTGATCTTGTGTGGACTGATAATTTGCGTGTGTTAAGCGCGTATGCGCATGAACGTTCATCTTCAACTCGGCTTTCTACGGAAGCG
>1_DWV-A-R_Worker Ants-20Oct2022_A03_2022-10-20_1.ab1 (101 bases) [100%; OP889268.1]
CACAAGATCAAAGTCAGCAATATGTTCATGACGCTTACTACACCACGTAGCTATATATTTCTTCTGTTTTCCAGTTATATAACCTTCATCCACAAGACAAA
>2_DWV-A-F_Worker Ants-20Oct2022_B01_2022-10-20_1.ab1 (111 bases) [98.17%; MF409157.1]
GTAAGCGTCATGAACATATTGCTGACTTTGATCTTGTGTGGACTGATAATTTGCGTGTGTTAAGCGCGTATGCGCATGAACGTTCATCTTCAACTCGGCTTTCTACGGAAG
>2_DWV-A-R_Worker Ants-20Oct2022_B03_2022-10-20_1.ab1 (120 bases) [99.15%; OP889268.1]
CACGCCAATTATCAGTCCACACAAGATCAAAGTCAGCAATATGTTCATGACGCTTACTACACCACGTAGCTATATATTTCTTCTGTTTTCCAGTTATATAACCTTCATCCACAAGACAAA
>3_DWV-A-F_Worker Ants-20Oct2022_C01_2022-10-20_1.ab1 (110 bases) [98.17%; MF409157.1]
GTAAGCGTCATGAACATATTGCTGACTTTGATCTTGTGTGGACTGATAATTTGCGTGTGTTAAGCGCGTATGCGCATGAACGTTCATCTTCAACTCGGCTTTCTACGGAA
>3_DWV-A-R_Worker Ants-20Oct2022_C03_2022-10-20_1.ab1 (108 bases) [100%; OP889268.1]
GTCCACACAAGATCAAAGTCAGCAATATGTTCATGACGCTTACTACACCACGTAGCTATATATTTCTTCTGTTTTCCAGTTATATAACCTTCATCCACAAGACAAAAC
>4_DWV-A-R_Worker Ants-20Oct2022_D03_2022-10-20_1.ab1 (112 bases) [100%; OP889268.1]
ATTATCAGTCCACACAAGATCAAAGTCAGCAATATGTTCATGACGCTTACTACACCACGTAGCTATATATTTCTTCTGTTTTCCAGTTATATAACCTTCATCCACAAGACAA
>5_DWV-A-F_Pupae-20Oct2022_E01_2022-10-20_1.ab1 (107 bases) [99.23%; MF409157.1]
GTCGATAGAACATATTGCTGACTTTGATCTTGTGTGGACTGATAATTTGCGTGTGTTAAGCGCGTATGCGCCATGAACGTTCATCTTCAACTCGGCTTTCTACGGAA
>6_DWV-A-F_Worker Ants-20Oct2022_F01_2022-10-20_1.ab1 (122 bases) [97.39%; MF409157.1]
CTACGGAGTAGTAAGCGTCATGAACATATTGCTGACTTTGATCTTGTGTGGACTGATAATTTGCGTGTGTTAAGCGCGTATGCGCATGAACGTTCATCTTCAACTCGGCTTTCTACGGAAAA
>6_DWV-A-R_Worker Ants-20Oct2022_F03_2022-10-20_1.ab1 (106 bases) [98.06%; OP889268.1]
TCCACAACAGGATCAAAGTCAGCAATATGTTCATGACGCTTACTACACCACGTAGCTATATATTTCTTCTGTTTTCCAGTTATATAACCTTCATCCACAAGACAGA
>7_DWV-A-F_Worker Ants-20Oct2022_G01_2022-10-20_1.ab1 (117 bases) [97.35%; MF409157.1]
GGATGTAGTAGCGTCATGAACATATTGCTGACTTTGATCTTGTGTGGACTGATAATTTGCGTGTGTTAAGCGCGTATGCGCATGAACGTTCATCTTCAACTCGGCTTTCTACGGAAA
>7_DWV-A-R_Worker Ants-20Oct2022_G03_2022-10-20_1.ab1 (100 bases) [98.92%; OP889268.1]
AAAAATCAAAGTCAGCAATATGTTTCATGACGCTTACTACACCACGTAGCTATATATTTCTTCTGTTTTCCAGTTATATAACCTTCATCCACAAGACAAA
>8_DWV-A-F_DW male Alates pooled-20Oct2022_H01_2022-10-20_1.ab1 (108 bases) [98.11%; MF409157.1]
TAGCGTCATGAACATATTGCTGACTTTGATCTTGTGTGGACTGATAATTTGCGTGTGTTAAGCGCGTATGCGCATGAACGTTCATCTTCAACTCGGCTTTCTACGGAA
>9_DWV-A-F_NW male Alates pooled-20Oct2022_A02_2022-10-20_1.ab1 (116 bases) [95.45%; MF409157.1]
TATGCGTCATGCAACATATTGCTGCACTTTGATCTTGTGTGGACTGATAATTTGCGTGTGTTAAGCGCGTATGCGCATGAACGTTCATCTTCAACTCGGCTTTCTACGGAACGGAA
>10_DWV-A-F_DW male Alate-20Oct2022_B02_2022-10-20_1.ab1 (112 bases) [97.30%; MF409157.1]

AGTAAGCGTCATCGAACATATTGCTGACTTTGATCTTGTGTGGACTGATAATTTGCGTGTGTTAAGCGCGTATGCGCATGAACGTTCATCTTCAACTCGGCTTTCTACGGAA
>10_DWV-A-R_DW male Alate-20Oct2022_B04_2022-10-20_1.ab1 **(112 bases) [98.18%; OP889268.1]**
TTATCAGCTCCACACCAGATCAAAGTCAGCAATATGTTCATGACGCTTACTACACCACGTAGCTATATATTTCTTCTGTTTTCCAGTTATATAACCTTCATCCACAAGACAA
>11_DWV-A-F_DW male Alate-20Oct2022_C02_2022-10-20_1.ab1 **(102 bases) [98.96%; MF409157.1]**
CGAGACATATTGCTGACTTTGATCTTGTGTGGACTGATAATTTGCGTGTGTTAAGCGCGTATGCGCATGAACGTTCATCTTCAACTCGGCTTTCTACGGAAA
>12_DWV-A-F_DW male Alate-20Oct2022_D02_2022-10-20_1.ab1 **(104 bases) [98.94%; MF409157.1]**
CTTCGAGAATATTGCTGACTTTGATCTTGTGTGGACTGATAATTTGCGTGTGTTAAGCGCGTATGCGCATGAACGTTCATCTTCAACTCGGCTTTCTACGGAAG
>14_DWV-A-F_DW male Alates pooled-20Oct2022_F02_2022-10-20_1.ab1 (111 bases) [98.17%; MF409157.1]
GTAAGCGTCATGAACATATTGCTGACTTTGATCTTGTGTGGACTGATAATTTGCGTGTGTTAAGCGCGTATGCGCATGAACGTTCATCTTCAACTCGGCTTTCTACGGAAA

>DWV-A Contig_1 (182 bases) [98.82%; MF409144.1]
GWaTTTGTCTTGTGGATGAAGGTTATATAACTGGAAAACAGAAGAAATATATAGCTACGTGGTGTAGTAAGCGTCATGAACATATTGCTGACTTTGATCTTGTGTGGACTGATAATTTGCGTGTGTTAAGCGCGTATGCGCATG
AACGTTCATCTTCAACTCGGCTTTCTACGGAARVGGAA

Supplementary Materials: Data Set S1D-F. Results of Sanger sequencing of multiple samples from pooled DW and NW male and female alates and pupae of *S. invicta* showing positive detection for DWV using subvariant-A-specific primers.

D

>1-DWV-A-F_DWV-pooled 1Nov22_A02_2022-11-01_2.ab1 **(114 bases) (male) [98.23%; MF409157.1]**
TGTAGTAAGCGTCATGAACATATTGCTGACTTTGATCTTGTGTGGACTGATAATTTGCGTGTGTTAAGCGCGTATGCGCATGAACGTTCATCTTCAACTCGGCTTTCTACGGAA
>1-DWV-A-R_DWV-pooled 1Nov22_A04_2022-11-01_2.ab1 **(107 bases) (male) [99.06%; OP889268.1]**
TCCACACAAGATCAAAGTCAGCAATATGTTCATGACGCTTACTACACCACGTAGCTATATATTTCTTCTGTTTTCCAGTTATATAACCTTCATCCACAAGACATTCG
>2-DWV-A-F_DWV-pooled 1Nov22_B02_2022-11-01_2.ab1 **(119 bases) (male) [97.41%; MF409157.1]**
CGATGGTGTAGTAGCGTCATGAACATATTGCTGACTTTGATCTTGTGTGGACTGATAATTTGCGTGTGTTAAGCGCGTATGCGCATGAACGTTCATCTTCAACTCGGCTTTCTACGGAA
>3-DWV-A-F_DWV-pooled 1Nov22_C02_2022-11-01_2.ab1 **(117 bases) (male) [97.37%; MF409157.1]**
TGCGTGTAGTAGCGTCATGAACATATTGCTGACTTTGATCTTGTGTGGACTGATAATTTGCGTGTGTTAAGCGCGTATGCGCATGAACGTTCATCTTCAACTCGGCTTTCTACGGAA
>3-DWV-A-F_DWV-pooled 1Nov22_C01_2022-11-01_2.ab1 **(110 bases) (male) [97.27%; MF409157.1]**
AGTAGCGTCATGAACATATTGCTGACTTTGATCTTGTGTGGACTGATAATTTGCGTGTGTTAAGCGCGTATGCGCATGAACGTTCATCTTCAACTCGGCTTTCTACGGAA
>3-DWV-A-R_DWV-pooled 1Nov22_C04_2022-11-01_2.ab1 **(112 bases) (male) [100%; OP889268.1]**
AATTATCAGTCCACACAAGATCAAAGTCAGCAATATGTTCATGACGCTTACTACACCACGTAGCTATATATTTCTTCTGTTTTCCAGTTATATAACCTTCATCCACAAGACA
>3-DWV-A-R_DWV-pooled 1Nov22_C03_2022-11-01_2.ab1 **(111 bases) (male) [100%; OP889268.1]**
ATTATCAGTCCACACAAGATCAAAGTCAGCAATATGTTCATGACGCTTACTACACCACGTAGCTATATATTTCTTCTGTTTTCCAGTTATATAACCTTCATCCACAAGACA
>4-DWV-A-F_DWV-pooled 1Nov22_D02_2022-11-01_2.ab1 **(125 bases) (male) [97.41%; MF409157.1]**
ATGGTGTAGTAGGCGTCATGAACATATTGCTGACTTTGATCTTGTGTGGACTGATAATTTGCGTGTGTTAAGCGCGTATGCGCATGAACGTTCATCTTCAACTCGGCTTTCTACGGAAATAACTG
>4-DWV-A-R_DWV-pooled 1Nov22_D04_2022-11-01_2.ab1 **(126 bases) (male) [100%; OP889268.1]**
CACGCAAATTATCAGTCCACACAAGATCAAAGTCAGCAATATGTTCATGACGCTTACTACACCACGTAGCTATATATTTCTTCTGTTTTCCAGTTATATAACCTTCATCCACAAGACAAGGGTGTT
>4-DWV-A-R_DWV-pooled 1Nov22_D03_2022-11-01_2.ab1 **(110 bases) (male) [100%; OP889268.1]**
TATCAGTCCACACAAGATCAAAGTCAGCAATATGTTCATGACGCTTACTACACCACGTAGCTATATATTTCTTCTGTTTTCCAGTTATATAACCTTCATCCACAAGACAA
>7-DWV-A-F_DWV-pooled 1Nov22_G02_2022-11-01_2.ab1 **(113 bases) (male) [96.26%; MF409157.1]**
CGTCGATGTAGTACGCGTCATGAACATATTGCTGACTTTGATCTTGTGTGGCACTGATAATTTGCGTGTGTTAAGCGCGTATGCGCATGAACGTTCATCTTCAACTCGGCTTT
>7-DWV-A-R_DWV-pooled 1Nov22_G04_2022-11-01_2.ab1 **(109 bases) (female) [99.04%; OP889268.1]**
AGTCCACACAGATCAAAGTCAGCAATATGTTCATGACGCTTACTACACCACGTAGCTATATATTTCTTCTGTTTTCCAGTTATATAACCTTCATCCACAAGACAAGCCA

>8-DWV-A-R_DWV-pooled 1Nov22_H04_2022-11-01_2.ab1 (102 bases) (female) [100%; OP889268.1]
CAAGATCAAAGTCAGCAATATGTTCATGACGCTTACTACACCACGTAGCTATATATTTCTTCTGTTTTCCAGTTATATAACCTTCATCCACAAGACAGGAGG

>Contig_1 (186 bases) [98.82%; MF409144.1]
CAGTTATTTCCGTAGAAAGCCGAGTTGAAGATGAACGTTCATGCGCATACGCGCTTAACACACGCAAATTATCAGTCCACACAAGATCAAAGTCAGCAATATGTTCATGACGCTTACTACACCACGTAGCTATATATTTCTTCTGT
TTTCCAGTTATATAACCTTCATCCACAAGACAAGSSWGTT

E

Pupa_DWV-A-F_DWV_male pupa-11-29-2022 (115 bases) [98.26%; MF409157.1]
GGTGTAGTAAGCGTCATGAACATATTGCTGACTTTGATCTTGTGTGGACTGATAATTTGCGTGTGTTAAGCGCGTATGCGCATGAACGTTCATCTTCAACTCGGCTTTCTACGGA
Pupa_DWV-A-R_DWV_male pupa-11-29-2022 (115 bases) [99.13%; OP889268.1]
CGCAATTATCAGTCCACACAAGATCAAAGTCAGCAATATGTTCATGACGCTTACTACACCACGTAGCTATATATTTCTTCTGTTTTCCAGTTATATAACCTTCATCCACAAGACA

>Contig_1_DW_male pupa-11-29-2022 (179 bases) [98.22%; MF409144.1]
AAGCCCGTTGTCTTGTGGATGAAGGTTATATAACTGGAAAACAGAAGAAATATATAGCTACGTGGTGTAGTAAGCGTCATGAACATATTGCTGACTTTGATCTTGTGTGGACTGATAATTtGCGTGTGTtAAGCGCGTAtGCGCM
TGAACGTTCATCT
TCAACTCGGCTTTCTACGGAA

F

>13_DWV-A-R_DWV-27Oct2022_E04_2022-10-27_1.seq (157 bases) (female) [98.26%; OP889268.1]
ACCGGGGCTCCAACACTTTTTTTGCTCGCAAACAAATTTATCAGTCCACACAAAGATCAAAGTCAGCAATATGTTCATGACGCTTACTACACCACGTAGCTATATATTTCTTCTGTTTTCCAGTTATATAACCTTCATCCACAAGACA
AACCCGTCG

Supplementary Materials: Data Set S1G. Results of Sanger sequencing of multiple samples from DW male and female alates, and workers, of *S. invicta* showing positive detection for the replicative form of DWV using primers specific for the RNA-dependent RNA polymerase (RD-RP) gene. Also, there are samples that were diluted two-times.

G

>15-Beehead_DWV-B23_DWV-rep-17Feb23_G04_2023-02-21_2.ab1 (424 bases) [100%; MG831204.1]
GGGAATACAATTTCAAATTGTTTGTTAATTAGGTTAGCTTGGTTAGGTATTACTGATTTGCCTTTGTCCGAGTTCTCTCAAAATGTTGTTCTTGTCTGTTATGGCGACGATCTTATCATGAATGTTAGCGATAACATGATTGATAAGT
TTAACGCCGT
GACGATAGGAAAATTCTTTTCACAATATAAGATGGAATTTACGGATCAGGATAAATCAGGAAATACTGTAAAGTGGCGGACGTTACAGACTGCTACTTTCTTAAAACATGGGTTCTTAAAACATCCAACTAGACCTGTGTTTCTA
GCTAACCTGGACA
AGGTCTCGGTAGAAGGAACGACGAATTGGACTCATGCTCGAGGATTGGGTCGTCGTACAGCAACCATAGAAAATGCTAAACAAGCGTTAGAGTTAGCATTTGGGTGGA
>15-Beehead_DWV-B23_2XD_DWV-rep-17Feb23_G08_2023-02-21_2.ab1 (two-times dilution) (401 bases) [99.74%; MH069505.1]
CACAATCCTAGAGCATGAGTCCAATTCGTCGTTCCTTCTACCGAGACCTTGTCCAGGTTAGCTAGAAACACAGGTCTAGTTGGATGTTTTAAGAACCCATGTTTTAAGAAAGTAGCAGTCTGTAACGTCCGCCACTTTACAGTATT
TCCTGATTTATC
CTGATCCGTAAATTCCATCTTATATTGTGAAAAGAATTTTCCTATCGTCACGGCGTTAAACTTATCAATCATGTTATCGCTAACATTCATGATAAGATCATCGCCATAACAGACAAGAACAACATTTTGAGAGAACTCGGACAAAG
GCAAATCAGTAA
TACCTAACCAAGCTAACCTAATTAACAAACAATTTGAAATTGTATTCAATATGTCCGTTATTGGAGAACCTGATGGACCACCCTA
>3-DW-F-DWV-B23_DWV-rep-17Feb23_C03_2023-02-21_1.ab1 (385 bases) (female) [97.80%; MG831204.1]

AGGGTGTTCTTCGTCTCCTTCGTCCGAATTCTCTCAAAATGGTTGGCTTGTCTGTTATGGCGACGATCTTATCATGAATGTTAGCGATAACATGATTGATAAGTTTAACGCCGTGACGATGGAAAATTCTTTTCACAATATAAGATG
GAATTTACGGA
TCAGGATAAATCAGGAAATACTGTAAAGTGGCGGACGTTACAGACTGCTACTTTCTTAAAACATGGGTTCTTAAAACATCCAACTAGACCTGTGTTTCTAGCTAACCTGGACAAGGTCTCGGTAGAAGGAACGACGAATTGGACT
CATGCTCGAGGAT
TGGGTCGTCGTACAGCAACCATAGAAAATGCTAAACAAGCGTTAGAGTTAGATTTTGGGTGGACACCGT

>3-DW-F-DWV-B23_2XD_DWV-rep-17Feb23_C07_2023-02-21_1.ab1 (two-times dilution) (370 bases) (female) [97.22%; MG831204.1]
TACCTTCTCCTAGACCTTGTTGAGTGAGCTGGAAACACAGGTTAAGTTGGATGTTTTAAGAACCCATGTTTTAAGAAAGTAGCAGTCTGTAACGTCCGCCACTTTACAGTATTTCCTGATTTATCCTGATCCGTAAATTCCATCTTAT
ATTGTGAAAA
GAATTTTCCTATCGTCACGGCGTTAAACTTATCAATCATGTTATCACTAACATTCATGATAAGATCGTCGCCATAACAGACAAGAACAACATTTTGAGAGAACTCGGACAAAGGCAAATCAGTAATACCTAACCAAGCTAACCTAA
TTAACAAACAAT
TTGAAATTGTATTCAATATGTCCGTTATTGGAGAACCTGATGGACCACGGGCGG

>DW-M_DWV-B23-14Feb2023-1_A02_2023-02-14_2.ab1 (275 bases) (male) [100%; MN902115.1]
AGCAGTCTGTAACGTCCGCCACTTTACAGTATTTCCTGATTTATCCTGATCCGTAAATTCCATCTTATATTGTGAAAAGAATTTTCCTATCGTCACGGCGTTAAACTTATCAATCATGTTATCACTAACATTCATGATAAGATCGTCG
CCATAACAGA
CAAGAACAACATTTTGAGAGAACTCGGACAAAGGCAAATCAGTAATACCTAACCAAGCTAACCTAATTAACAAACAATTTGAAATTGTATTCAATATGTCCGTTATTGGAGAACCTG

>DW-M-Col14_DWV-B23-14Feb2023-1_E02_2023-02-14_1.ab1 (194 bases) (male) [99.48%; KP734594.1]
TCCTCGAGCATGAGTCCAATTCGTCGTTCCTTCTACCGAGACCTTGTCCAGGTTAGCTAGAAACACAGGTCTAGTTGGATGTTTTAAGAACCCATGTTTTAAGAAAGTAGCAGTCTGTAACGTTCGCCACTTTACAGTGTTTCCTGA
TTTATCCTGAT
CCGTAAATTCCATCTTATATTGTGAAAAGAATTTTC

>12-Workers-Col14_DWV-B23_DWV-rep-17Feb23_D04_2023-02-21_1.ab1 (347 bases) [99.42%; MT068461.1]
TCTCAAAAGGTTGTTCTTGTCTGTTATGGCGACGATCTTATCATGAATGTTAGTGATAACATGATTGATAAGTTTAACGCCGTGACGATAGGAAAATTCTTTTCACAATATAAGATGGAATTTACGGATCAGGATAAATCAGGAAA
TACTGTAAAGTG
GCGGACGTTACAGACTGCTACTTTCTTAAAACATGGGTTCTTAAAACATCCAACTAGACCTGTGTTTCTAGCTAACCTGGACAAGGTCTCGGTAGAAGGAACGACGAATTGGACTCATGCTCGAGGATTGGGTCGTCGTACAGCA
ACCATAGAAAATG
CTAAACAAGCGTTAGAGTTAGCATTTGGGTG

Supplementary Materials: Data Set S1H. Results of Sanger sequencing of multiple samples from DW male and female alates, and workers, of *S. invicta* showing positive detection for the replicative form of DWV using a tag-primer that was incorporated into a fragment of the RNA-dependent RNA polymerase (RD-RP) gene during the reverse transcriptase step. Also, there are samples diluted two-times.

H

>15-Beehead_Tag_DWV-rep-17Feb23_G02_2023-02-21_2.ab1 (411 bases) [99.26%; MW222481.1]
TGTGTGTTAATTAGGTTAGCTTGGTTAGGTATTACTGATTTGCCTTTGTCCGAGTTCTCTCAAAATGTTGTTCTTGTCTGTTATGGCGATGATCTTATCATGAATGTTAGCGATAACATGATTGATAAGTTTAACGCCGTGACGATA
GGAAAATTCTT
TTCACAATATAAGATGGAATTTACGGATCAGGATAAATCAGGAAATACTGTAAAGTGGCGGACGTTACAGACTGCTACTTTCTTAAAACATGGGTTCTTAAAACATCCAACTAGACCTGTGTTTCTAGCTAACCTGGACAAGGTC
TCGGTAGAAGGAA
CGACGAATTGGACTCATGCTCGAGGATTGGGTCGTCGTACAGCAACCATAGAAAATGCTAAACAAGCGTTAGAGTTAGCATTTTGGGGTGGAGAT
>15-Beehead_Tag_2XD_DWV-rep-17Feb23_G06_2023-02-21_2.ab1 (two-times dilution) (381 bases) [97.88%; MG831204.1]
AGCTTTGGTTAGGTATTACCGATTTGCCTTTGACAGAGTTCTCTCTAATGTTGTTCTTGTCTGTTATGGCGACGATCTTATCATGAATGTTAGTGATAACATGATTGATAAGTTCAACGCCGTGACGATAGGAAAATTCTTTTCACA
ATATAAGATGG
AATTTACGGATCAGGATAAATCAGGAAATACTGTAAAGTGGCGGACGTTACAGACTGCTACTTTCTTAAAACATGGGTTCTTAAAACATCCAACTAGACCTGTGTTTCTAGCTAACCTGGACAAGGTCTCGGTAGAAGGAACGAC
GAATTGGACTCAT
GCTCGAGGATTGGGTCGTCGTACAGCAACCATAGAAAATGCTAAACAAGCGTTAGAGTTAGTTTT
>NW-M-B1_Tag_DWV-rep-16Feb23_F01_2023-02-16_3.ab1 (414 bases) (male) [98.77%; MG831204.1]
TTGTTACATTTCAATTGTTTGTTAATTAGGTTAGCTTGGTTAGGTATTACTGATTTGCCTTTGTCCGAGTTCTCTCAAAATGTTGTTCTTGTCTGTTATGGCGACGATCTTATCATGAATGTTAGTGATAACATGATTGATAAGTTCAA
CGCCGTGAC
GATAGGAAAATTCTTTTCACAATATAAGATGGAATTTACGGATCAGGATAAATCAGGAAATACTGTAAAGTGGCGGACGTTACAGACTGCTACTTTCTTAAAACATGGGTTCTTAAAACATCCAACTAGACCTGTGTTTCTAGCTA
ACCTGGACAAGG
TCTCGGTAGAAGGAACGACGAATTGGACTCATGCTCGAGGATTGGGTCGTCGTACAGCAATCCATAGAAAATGCTAAACAAGCGTTAGAGTCTTTTTT
>14-NW-M-1B_Tag_2XD_DWV-rep-17Feb23_F06_2023-02-21_2.ab1 (two-times dilution) (373 bases) (male)[98.66%; MG831204.1]
ATGGTTGGGTATTACTGATTTGCCTTTGTCAGAGTTCTCTTAAAATGTTGTTCTTGTCTGTTATGGCGACGATCTTATCATGAATGTTAGTGATAACATGATTGATAAGTTCAACGCCGTGACGATAGGAAAATTCTTTTCACAATA
TAAGATGGAAT
TTACGGATCAGGATAAATCAGGAAATACTGTAAAGTGGCGGACGTTACAGACTGCTACTTTCTTAAAACATGGGTTCTTAAAACATCCAACTAGACCTGTGTTTCTAGCTAACCTGGACAAGGTCTCGGTAGAAGGAACGACGA
ATTGGACTCATGCT
CGAGGATTGGGTCGTCGTACAGCAACCATAGAAAATGCTAAACAAGCGTTAGAGTTA
>4-DW-F-Tag_DWV-rep-17Feb23_D01_2023-02-21_1.ab1 (344 bases) (female) [98.55%; MT068461.1]
TCTCTCAAAATGTTGTTCTTGTCTGTTATGGCGACCATCTTATCATGAATGTTAGTGATAACATGATTGATAAGTTCAACGCCGTGACGATGGGAAAATTCTTTTCACAATATAAGATGGAATTTACGGATCAGGATAAATCAGGA
AATACTGTAAAG
TGGCGGACGTTACAGACTGCTACTTTCTTAAAACATGGGTTCTTAAAACATCCAACTAGACCTGTGTTTCTAGCTAACCTGGACAAGGTCTCGGTAGAAGGAACGACGAATTGGACTCATGCTCGAGGATTGGGTCGTCGTACAG
CAACCATAGAAAA
TGCTAAACAAGCGTTAGAGTTAGATTTT
>4-DW-F-Tag_2XD_DWV-rep-17Feb23_D05_2023-02-21_1.ab1 (two-time dilution) (377 bases) (female) [97.05%; MG831204.1]
AGACTGATTTGAGCTTGTGAGTGTTCTCTCGGATGGTGTTCTTGTCTGTTATGGCGACGATCTTATCATGAATGTTAGCGATAACATGATTGATAAGTTTAACGCCGTGACGATAGGAAAATTCTTTTCACAATATAAGATGGAAT
TTACGGATCAGG
ATAAATCAGGAAATACTGTAAAGTGGCGGACGTTACAGACTGCTACTTTCTTAAAACATGGGTTCTTAAAACATCCAACTAGACCTGTGTTTCTAGCTAACCTGGACAAGGTCTCGGTAGAAGGAACGACGAATTGGACTCATGC
TCGAGGATTGGGT
CGTCGTACAGCAACCATAGAAAATGCTAAACAAGCGTTAGAGTTAGCATTTTGGGTGGAGA
>9-DW-M-Col8_Tag_DWV-rep-17Feb23_A06_2023-02-21_1.ab1 (413 bases) (male) [99.51%; MG831204.1]

ATACATTTCAATTGTTTGTTAATTAGGTTAGCTTGGTTAGGTATTACTGATTTGCCTTTGTCCGAGTTCTCTCAAAATGTTGTTCTTGTCTGTTATGGCGACGATCTTATCATGAATGTTAGCGATAACATGATTGATAAGTTTAACG
CCGTGACGAT
AGGAAAATTCTTTTCACAATATAAGATGGAATTTACGGATCAGGATAAATCAGGAAATACTGTAAAGTGGCGGACGTTACAGACTGCTACTTTCTTAAAACATGGGTTCTTAAAACATCCAACTAGACCTGTGTTTCTAGCTAACC
TGGACAAGGTCT
CGGTAGAAGGAACGACGAATTGGACTCATGCTCGAGGATTGGGTCGTCGTACAGCAACCATAGAAAATGCTAAACAAGCGTTAGAGTTAGTTTTTTG

>Workers-Col14_Tag_DWV-rep-16Feb23_D01_2023-02-16_3.ab1 (426 bases) [97.58%; MG831204.1]

GGGGCGTTAGAGTTAGCATTTGGGTGGAGGTTAGGCAAGCTTTCCTAGGCAGACTGATTTGACCTTGTGAGTGTTCTCTCGGATGGTGTTCTTGTCTGTTATGGCGACGATCTTATCATGAATGTTAGCGATAACATGATTGATA
AGTTTAACGCCGT
GACGATAGGAAAATTCTTTTCACAATATAAGATGGAATTTACGGATCAGGATAAATCAGGAAATACTGTAAAGTGGCGGACGTTACAGACTGCTACTTTCTTAAAACATGGGTTCTTAAAACATCCAACTAGACCTGTGTTTCTA
GCTAACCTGGACA
AGGTCTCGGTAGAAGGAACGACGAATTGGACTCATGCTCGAGGATTGGGTCGTCGTACAGCAACCATAGAAAATGCTAAACAAGCGTTAGAGTTAGCATTTGGGTGGAAA

Supplementary Materials: Data Set S1.

In the above sequencing results, (**A**) Results of Sanger sequencing of samples of *S. invicta* ants from Figure 3., showing positive results for replicative form of DWV: adult workers from colony 8, (pooled, *n*=10), worker pupae, colony 10, (pooled, *n*=5), and deformed wing (DW) male alates, colony 8, (pooled, *n* = 5), showing positive detection for the replicative form of DWV using a tag-primer that was incorporated into a fragment of the RNA-dependent RNA polymerase (RD-RP) gene during the reverse transcriptase step. Samples were ten-times diluted. Additional sequence is from a NW male alates showing the replicative form of the DWV, which is at two-times dilution. (**B**) are results for Sanger sequencing showing the identification of DWV-A to be present in *S. invicta* workers from the following colonies read assignments are as follows: 1.) Col. 2021-worker ants-DWV-A, 2.) Col. B1-worker ants-DWV-A, 3.) Col. 14-worker ants-DWV-A, 4.) Col. 8-pupae-DWV-A, 6.) Col. Greenville-1-worker ants-DWV-A, 7.) Honeybee DWV (+) control. Also have a queen from Greenville-1 positive for DWV, and a NW female re-run positive for DWV. (**C**), are results for Sanger sequencing showing the identification of DWV-A to be present in *S. invicta* workers and male and female alates with and without wing deformity. Read assignments are as follows: 1.) Col. 2021-worker ants-DWV-A, 2.) Col. B1-worker ants-DWV-A, 3.) Col. 14-worker ants-DWV-A, 4.) Col. 8-worker ants-DWV-A, 5.) Col. 8-pupae-DWV-A, 6.) Col. Greenville-1-worker ants-DWV-A, 7.) Col. Greenville-2 worker ants-DWV-A, 8.) DW-male alates (*n*=5)-DWV-A, 9.) NW male alates (*n*=5)-DWV-A, 10.) Col. 11-DW-male alate-DWV-A, 11.) Col. NCBL-DW-male alate-DWV-A, 12.) Col. 9-DW-male alate-DWV-A, 14.) DW-male-pooled-DMV-A. (**D**), are Sanger sequencing detecting DWV-A in additional *S. invicta* male and female alates. Read assignments are as follows: 1-2.) Col. 4-DW male alates-DWV-A (*n*=4), 3-4.) Col. 4-NW male alates-DWV-A (*n*=4), 7-8.) Col. NBCL-7-NW female alates (*n*=4). All samples were pooled. Contig_1. (**E**), are results for Sanger sequencing showing the identification of DWV-A to be present in a non-melanized pupa of *S. invicta*. (**F**), are results for a re-run of a female DW alate due to original sequencing issues experienced on 20-Oct-2022. (**G**), are results for Sanger sequencing showing the detection of replicative form of DWV in

various castes of *S. invicta*. Target gene is the RD-RP gene. Read assignments are as follows: 1-2.) Western honey bee (+ for DWV) *Apis mellifera*, 3) DW female, 4) DW female alate two-times dilution, 5) DW male alate, 6) Col. 14 DW male alate, 7) Col. 14 workers. (**H**) are results for tag-only primer used to sequence replicative form of DWV. Read assignments are as follows: 1-2) Western honey bee (+ for DWV) *Apis mellifera*, 3) NW male alate, 4) NW male alate two-times dilution, 5) DW female alate, 6) DW female alate two-times dilution, 7) Col. 8 DW male alate, 8) Col.14 workers.

Details for DNA gel electrophoresis results for *S. invicta*. All samples where there was an observed gel band corresponding to the positive control were sent off for Sanger sequencing. Not all sequencing worked; however, all samples that gave a readable sequence when blasted on NCBI, matched DWV. We used 5 µl of PCR product for DNA gel electrophoresis and, for DWV-A (PCR product size = 168 bp), 10 µl purified for Sanger sequencing. For worker ants (20 to 50 mg, fresh weight) and whole alates (single alate, 5 to 11 mg, fresh weight, or pooled, 30 to 50 mg), fresh weight. For the positive control, we used RNA from a Western honey bee with DW (+ for DWV) *Apis mellifera*. All no template controls (NTC) were negative for bands. For replicative form, all controls: NTC, no primers, and no transcriptase, were negative for bands.

Videos. Detailed description of eight videos depicting both deformed wing (DW) and normal wing (NW) male and female alates from numerous *S. invicta* lab and field colonies. The purposes for these videos are to let the reader observe the gross morphological and mobility differences between DW and NW alates. All videos were captured using a Keyence VHX 5000 (Itasca, IL), except for video 4, where a Samsung Galaxy J7 Star mobile phone, was used.

Video 1 Two deceased and one live DW male alate from *S.invicta* colony NBCL-2, which were collected directly from the *in situ* mound. Ants were collected the day before imaging. At the time of collecting and approximately 90 minutes before video capture, all three alates were alive and possessed some level of mobility but demonstrated an ataxic gait with difficulty righting themselves. All three alates have severe DW phenotype. The runtime for this video is 20 seconds.

Video 2 Two DW male alates and one NW male alate from Colony 8. The two DW alates are unable to right themselves. The alate, bottom left, died shortly after this video was taken. The alate on the right couldn't walk due to severely deformed legs. This is not a common deformity among the DW males thus far observed. The runtime for this video is 20 seconds.

Video 3 A DW male alate collected from colony 12 displaying severely deformed wings and an inability to walk. The alate's left back leg is partially paralyzed. Approximately three hours after video capture, the alate died. The runtime for this video is 25 seconds.

Video 4 Three DW male alates from colony 14. Two DW alates display severe leg deformity and are unable to stand or walk while the third, with slight wing deformity, displays mobility issues with its deformed left hind leg. The runtime for this video is 5 seconds.

Video 5 A cluster of DW male alates from colony 8 displaying various degrees of wing deformity. The runtime for this video is 20 seconds.

Video 6 A single DW male alates from colony 1. This alate has severely deformed wings and legs and is unable to right itself. The runtime for this video is 30 seconds.

Video 7 A single DW male alates from colony 3C. This alate has severely deformed wings and is unable to fully use its hind legs and, at a slower frame speed, one can see this alate is unable to fully extend its right front leg. The runtime for this video is 30 seconds.

Video 8 Two DW female alates – one with slight wing deformity and the other with severe wing deformity from Colony NBCL-12. Neither female alate shows any ataxia or paralysis. The runtime for this video is 30 seconds.

Reference

[34] Chen, J.; Du, Y. Fire ants feed their nestmates with their own venom. *J. Insect Physiol.* 2022, *142*: 104437 doi:10.1016/j.jinsphys.2022.104437

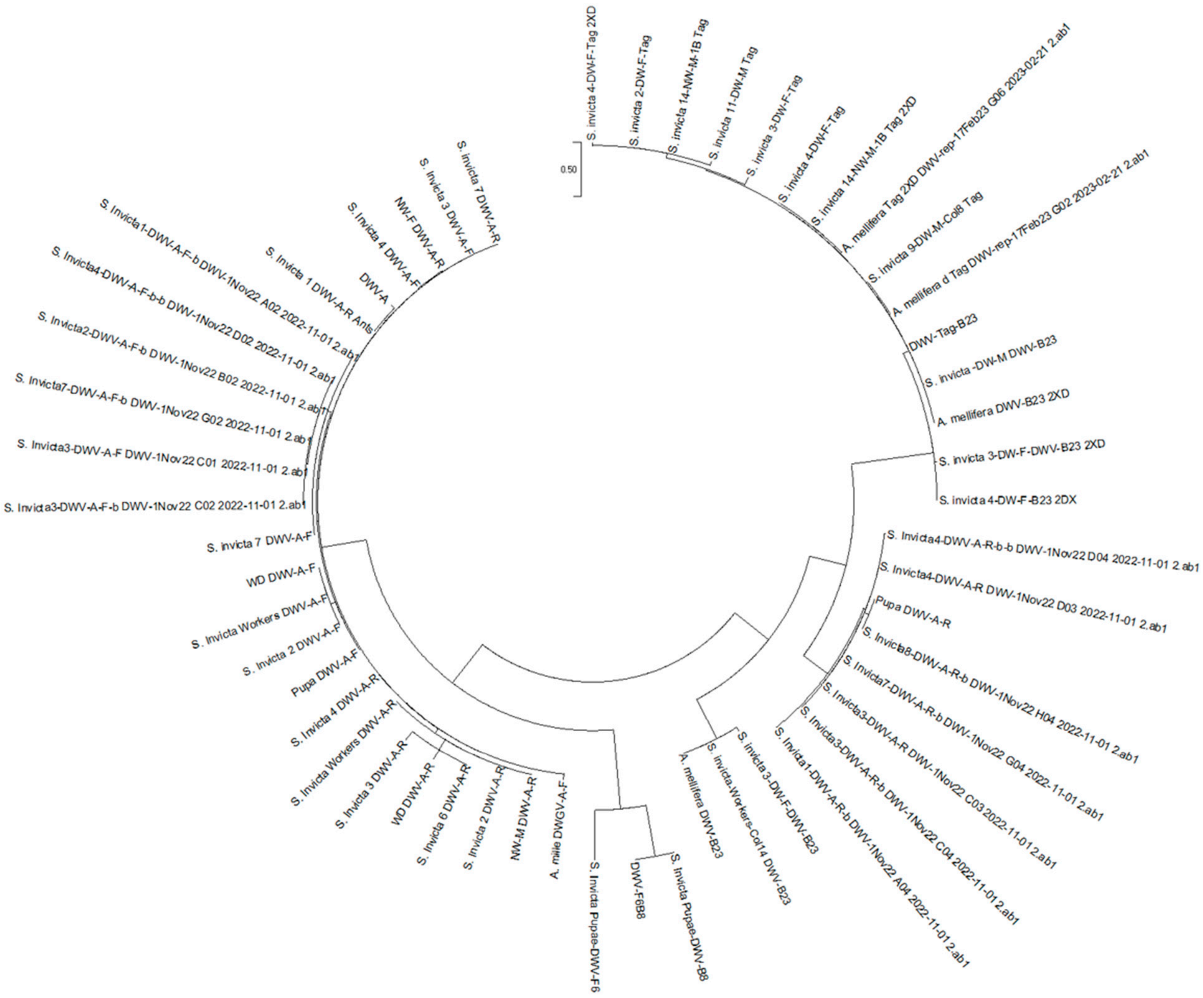

Figure S1. Phylogenetic tree based on sequences from samples of *S. Invicta* and positive control of *A. mellifera* with DWV against deformed wing virus sequence fragments on which the sequencing primer sets were located.
